# Normative brain mapping of interictal intracranial EEG to localize epileptogenic tissue

**DOI:** 10.1093/brain/awab380

**Published:** 2022-01-24

**Authors:** Peter N Taylor, Christoforos A Papasavvas, Thomas W Owen, Gabrielle M Schroeder, Frances E Hutchings, Fahmida A Chowdhury, Beate Diehl, John S Duncan, Andrew W McEvoy, Anna Miserocchi, Jane de Tisi, Sjoerd B Vos, Matthew C Walker, Yujiang Wang

**Affiliations:** 1 CNNP Laboratory (www.cnnp-lab.com), Interdisciplinary Computing and Complex BioSystems Group, School of Computing, Newcastle Helix, Newcastle University, Newcastle-upon-Tyne, NE4 5TG, UK; 2 UCL Queen Square Institute of Neurology and National Hospital for Neurology and Neurosurgery (NHNN), Queen Square, London WC1N 3BG, UK

**Keywords:** cortical localization, EEG, epilepsy surgery, epileptogenic zone, intracranial electrodes

## Abstract

The identification of abnormal electrographic activity is important in a wide range of neurological disorders, including epilepsy for localizing epileptogenic tissue. However, this identification may be challenging during non-seizure (interictal) periods, especially if abnormalities are subtle compared to the repertoire of possible healthy brain dynamics. Here, we investigate if such interictal abnormalities become more salient by quantitatively accounting for the range of healthy brain dynamics in a location-specific manner.

To this end, we constructed a normative map of brain dynamics, in terms of relative band power, from interictal intracranial recordings from 234 participants (21 598 electrode contacts). We then compared interictal recordings from 62 patients with epilepsy to the normative map to identify abnormal regions. We proposed that if the most abnormal regions were spared by surgery, then patients would be more likely to experience continued seizures postoperatively.

We first confirmed that the spatial variations of band power in the normative map across brain regions were consistent with healthy variations reported in the literature. Second, when accounting for the normative variations, regions that were spared by surgery were more abnormal than those resected only in patients with persistent postoperative seizures (*t* = −3.6, *P* = 0.0003), confirming our hypothesis. Third, we found that this effect discriminated patient outcomes (area under curve 0.75 *P* = 0.0003).

Normative mapping is a well-established practice in neuroscientific research. Our study suggests that this approach is feasible to detect interictal abnormalities in intracranial EEG, and of potential clinical value to identify pathological tissue in epilepsy. Finally, we make our normative intracranial map publicly available to facilitate future investigations in epilepsy and beyond.

## Teaser

Taylor *et al*. construct a normative map of brain dynamics from interictal intracranial recordings outside the seizure onset and initial propagation zones in >200 patients with epilepsy. They then leverage this map in a separate patient cohort to successfully identify epileptogenic tissue and predict surgical outcomes.

## Introduction

Abnormal electrographic activity is a hallmark of many neurological disorders. In focal epilepsy, ictal (seizure) periods commonly display clear pathological dynamics, which is clinically used to localize epileptogenic tissue. However, studies have suggested that interictal dynamics may also hold useful complementary information to identify epileptogenic tissue. For example, interictal spikes, sharp waves and high-frequency oscillations have all been suggested as putative markers.^1-8^

Grossly abnormal interictal events, such as interictal spikes, can often be identified visually or algorithmically. However, existing techniques may struggle to distinguish more subtle aberrations from the vast repertoire of possible healthy brain dynamics. Example healthy brain dynamics include beta oscillations, commonly seen in motor areas,^9,^^10^ and gamma activity in occipital and temporal areas.^11,^^12^ Other spatial profiles include alpha oscillations in occipital and parietal areas,^11,^^13^ delta in the temporal lobe^12–14^ and theta in superior frontal areas.^15,^^16^ In this work, we suggest that neural activity in these frequencies may also represent pathological activity if it occurs in brain regions that do not normally feature these frequencies. Conversely, a lack of power in typical frequencies of a particular brain region may also indicate pathological activity. Thus, identifying such subtle pathological activities requires the consideration of the spatial distribution of ‘normal’ electrographic activity.

One approach to account for normal spatial variations is to construct a normative map, which describes the healthy spatial profile and ranges of the feature of interest (in this case, the band power of different frequency bands). Such an approach is common and well-accepted in neuroimaging of brain structural abnormalities: patients are often normalized against healthy controls to highlight abnormal brain morphology^17^ or connectivity.^18,^^19^ However, for invasive recordings using intracranial EEG, data from healthy controls are not available. Instead, recent studies suggested using intracranial EEG recorded from areas outside of the putative seizure-generating tissue in patients with epilepsy.^13,^^20^ Specifically, Frauscher *et al*.^13^ conclude that this approach yields a normative map of brain dynamics that is consistent with data from animal models and other recording modalities. Owen *et al*.^21^ also motivated the use of normative maps using intracranial EEG.

In this study, we therefore follow this proposed approach to generate a normative map of band power across the brain using intracranial recordings from 234 participants with 21 598 recording contacts from outside the seizure onset and initial propagation zone. We first quantify the spatial distributions of normative band power and confirm agreement with previous data. Then, using a separate cohort of 62 patients with epilepsy, we show that accounting for the normative map allows us to identify epileptogenic tissue and subsequently predict patient surgical outcomes.

## Materials and methods

### Patients

Two main cohorts were studied here. The RAM normative cohort consisted of 234 participants with epilepsy undergoing presurgical evaluation with intracranial EEG to localize seizure onset. As part of the intracranial EEG monitoring, the participants were also participating in an experimental study on memory (data collected up to Year 3; http://memory.psych.upenn.edu/RAM). As stated in the project’s website ‘Informed consent has been obtained from each participant to share their data, and personally identifiable information has been removed to protect participant confidentiality’. The original research protocol for data acquisition was approved by the relevant bodies at the participating institutions. Furthermore, the University Ethics Committee at Newcastle University approved the analysis of this dataset (ref. 12721/2018). The normative recordings were obtained in the preparatory phase, several minutes before a memory task.

The UCLH epilepsy cohort consisted of 62 patients with epilepsy undergoing presurgical evaluation with invasive intracranial EEG to localize seizure onset. All patients had presurgical, pre-implantation (T_1_-weighted) MRI. All patients had either CT or T_1_-weighted MRI while implanted electrodes were in place. Most patients had postoperative T_1_-weighted MRI (*n* = 61). For the single patient without postoperative MRI, the detailed surgery report described the brain areas resected. At a follow-up of 12 months, 33 patients were free of disabling seizures and 29 had persistent seizures. Follow-up outcomes were defined as described previously according to the ILAE classification.^22^ A subset of this cohort has been studied previously.^23^ All data were anonymized and exported, then analysed under the approval of the Newcastle University Ethics Committee (2225/2017). Detailed patient metadata are shown in Supplementary material and summarized in Table 1. No significant differences were present between outcome groups in age, sex, lobe of resection, side of resection or number of electrode contacts.

**Table 1:** Summary of patient data

	ILAE1,2	ILAE3+	Test statistic
*n* (%)	33 (53)	29 (47)	
Age, mean (SD)	32.3 (10.7)	33.0 (8.8)	*P* = 0.8017, *t* = −0.2522
Sex, male: female	15:18	17:12	*P* = 0.3, χ^2^ = 1.07
Temporal, extratemporal	21, 12	15, 14	*P* = 0.34, χ^2^ = 0.8995
Side, left/right	18/15	16/13	*P* = 0.96, χ^2^ = 0.00024
Number of electrode contacts, mean (SD)	71.1 (24.3)	65.9 (23.3)	*P* = 0.3984, *t* = 0.8505

### MRI processing for electrode localization and resection delineation

Electrode contacts for all participants were localized to regions of interest defined according to a parcellation. To ensure robustness of our findings we investigated four separate parcellations at different resolutions where higher resolutions are subdivisions of lower resolutions. These parcellations have been described previously^24^ and have been used for normative intracranial analysis.^20^ Due to different levels of available data, our technique for localization of electrode contacts to regions differed slightly between the RAM and UCLH datasets. In the RAM data, electrode contact locations are publicly available as Talairach space coordinates, which we converted to MNI space.^25^ We next reconstructed an MNI space brain using FreeSurfer, matched each of the four parcellations to that surface using mri_surf2surf, obtained the labels and matched each contact to the closest volumetric region of interest (minimum Euclidean distance using custom code in MATLAB). For UCLH data, we performed broadly the same procedure but conducted the processing in native space. Performing native space processing was possible as the preoperative T_1_-weighted MRI was available along with the CT/MRI scan to mark electrode contacts as described previously.^23,^^26^ To identify which regions were removed/spared by surgery, we linearly registered the postoperative T_1_-weighted scan to the preoperative scan and manually delineated the resected tissue as a mask described previously.^23,^^27^ Electrode contacts were defined as removed if they were within 5 mm of the mask as in our previous work.^23^ In each patient, regions were defined as removed if >25% of contacts within the region were removed; otherwise, regions were considered spared.

### Intracranial EEG data and processing

To create a normative baseline of intracranial EEG (iEEG) spectral properties we used the RAM dataset, and extracted 70 s of iEEG recording from relaxed wakefulness (shortly before a memory task) for each participant. We excluded channels that were labelled as seizure onset zone, early propagation zone, brain lesions or bad contacts. The extracted EEG signals from the remaining channels were visually inspected for recording artefacts, and recording channels located in white matter were also excluded, resulting in a final set of 21 598 channels across 234 participants.

We further used a separate iEEG dataset from UCLH to compare and score against the normative baseline. Again, we retrospectively extracted 70 s of interictal iEEG recording for each participant, at least 2 h away from seizures. Where possible, the recording was obtained at around 2 p.m. in the afternoon to maximize the likelihood of wakefulness. Due to the retrospective design, it was not possible to determine the exact brain state. To demonstrate robustness, we also present results for two further time segments at least 2 h away from seizures and 4 h away from other time segments at around 9 a.m. and 7 p.m. where possible. For the UCLH dataset, we included all grey matter channels (i.e. even those in seizure onset zone, propagation zone and irritative zones). We only excluded artefactual channels and recording channels in white matter, resulting in 4256 channels across 62 patients.

All EEGs were downsampled to 200 Hz for the RAM dataset before creating the normative map. In the UCLH data, we had a mixture of sampling frequencies (two participants at 256 Hz, 52 participants at 512 Hz, eight participants at 1024 Hz and one participant at 2048 Hz). After applying a common average reference to all recordings in all participants, we estimated the power spectral density with Welch’s method (2 s window, 1 s overlap, Hamming window) in each 70 s recording. The average band power within five frequency bands of interest were then calculated using the ‘bandpower’ function in MATLAB. The following ranges were defined, delta (δ 1–4 Hz), theta (θ 4–8 Hz), alpha (α 8–13 Hz), beta (β 13–30 Hz) and gamma (γ 30–80 Hz). In the gamma band, data between 47.5 and 52.5 Hz, and 57.5 and 62.5 Hz were excluded to avoid power line artefacts in both the US and UK recordings. Band power estimates were then log${\;}_{10}$ transformed and normalized to sum to one for each contact (i.e. L1 norm). These transformed and normalized values represent the relative band power used throughout the results. Each participant therefore has a value of relative band power assigned to each contact and each frequency band.

For the UCLH dataset, the clinical team additionally provided information on whether any channels displayed interictal spikes (at any point during the recording). This information was used later as a baseline measure, and to demonstrate robustness of our results.

### Normative map generation

To obtain a normative distribution of relative band power in a particular frequency band and brain region, we first assigned each electrode contact from each participant in the RAM dataset to a grey matter region, as described before. One contact can only be assigned to a single (nearest) region. If multiple contacts from the same participant were assigned to the same region, then we averaged the relative band powers to obtain single values of relative band power per region and frequency band per patient. If zero contacts were assigned to a region in a particular participant, then the region was considered to have no coverage and the relative band powers were set to ‘not a number’ for that participant and region. The normative distribution of relative band power in a region (in a particular frequency band) was then obtained as the distribution of relative band powers of all RAM participants with coverage in that region. Coverage obtained in the normative map can be found in the Supplementary material.

**Figure 1:**
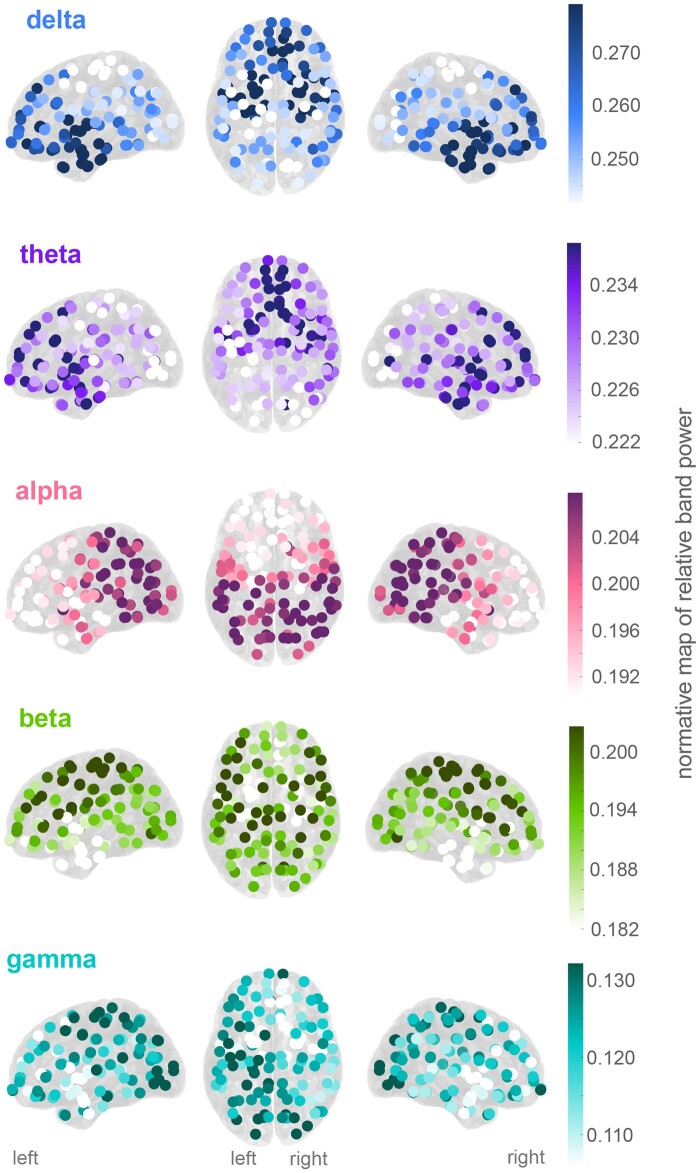
**Normative band power varies across regions.** Mean relative band power in each region for each of the five frequency bands of interest. The colour axes scale differs for each frequency band with generally higher power in lower frequencies.

To visualize the normative map, we plotted the mean of the distribution of relative band powers in a particular region and frequency band across normative participants (Fig. 1).

### Scoring patients to the normative map

To score the UCLH patient cohort against the normative map, we followed a similar approach in mapping the electrodes to brain regions. Electrode contacts for a given patient were localized to a single brain region (i). Where multiple contacts localized to the same region the mean band power value across contacts was used. This allows estimation of the band power in a given region (i), in a given frequency band (j), for a given patient. To estimate the abnormality of a region’s relative band power in the UCLH dataset from the normative map we computed the absolute *z*-score Equation (1):

(1) $$\left|z_{i,j}\right|=\left|\frac{x_{i,j}-\mu_{i,j}}{\sigma_{i,j}}\right|$$

where i represents the region, j the frequency band of interest, x is the band power value for an individual patient and μ and σ are the mean and standard deviations of the band powers in the normative map.

In comparing the values between resected and spared regions for any patient in the UCLH dataset and frequency band, we used the distinguishability statistic ($D_{RS}$), which is the area under the receiver operating curve, and equivalent to the normalized Mann–Whitney U-statistic (see Wang *et al*.,^23^ Ramaraju *et al*.^28^ and Bernabei *et al*.^29^). A $D_{RS}$ value >0.5 indicates that spared regions were more abnormal (higher absolute *z*-score) than resected regions, whereas $D_{RS}$ values <0.5 indicates the opposite—i.e. resected regions were more abnormal.

### Statistical analysis

Proposing that resected regions would be more abnormal than spared regions in good outcome patients, we tested for $D_{RS}$ <0.5 in outcome patients using a left-tailed one-sample *t*-test. In contrast, we hypothesized the opposite effect in poor outcome patients and tested $D_{RS}$ >0.5 using a one-sample right-tailed *t*-test. Finally, we hypothesized greater $D_{RS}$ values in poor outcome patients than good outcome patients, and tested with a two-sample left-tailed *t*-test.

Statistical significance is reported for *P* < 0.05 for reference. Effect sizes are reported throughout as *t*-statistics or as area under the receiver operating characteristic curve (AUC).

### Data and code availability

Preprocessed data and analysis code are available at the following link https://doi.org/10.5281/zenodo.5500400.

## Results

### Normative maps show spatial organization of band power

We constructed normative maps of relative power in five frequency bands (δ 1–4, θ 4–8, α 8–13, β 13–30 and γ 30–80 Hz). To construct the normative maps, we used 70 s of interictal intracranial EEG recordings from 21 598 electrode contacts outside of the seizure onset and initial propagation zone across 234 participants. The 70-s segments were recorded while the participants were awake and preparing for a cognitive task experiment. We derived the relative band power for five main frequency bands in all contacts. Each contact was then assigned to one of 128 regions of interest from the Lausanne scale60 atlas,^24^ yielding a normative distribution of relative band power in each region of interest.

The resulting normative maps of the mean relative band power for each frequency band are shown in Fig. 1A. Several distinct patterns can be observed; for example, relative delta power is most prominent in the anterior temporal and anterior frontal regions, while relative alpha power is prominent in parietal and occipital regions. Note that lower frequencies generally have higher relative power (the colour axes scale differs for each frequency band in Fig. 1A). Finally, the overall gradient of the normative maps also displays a striking symmetry between the left and right hemispheres. These normative spatial profiles are further quantified in Supplementary material.

### Normative maps highlight abnormalities in individual patients

We then turned our attention to a cohort of patients from UCLH with refractory focal epilepsy who underwent presurgical evaluation with intracranial EEG. We used the normative maps as a baseline to identify aberrations in each region of interest for individual patients.

**Figure 2:**
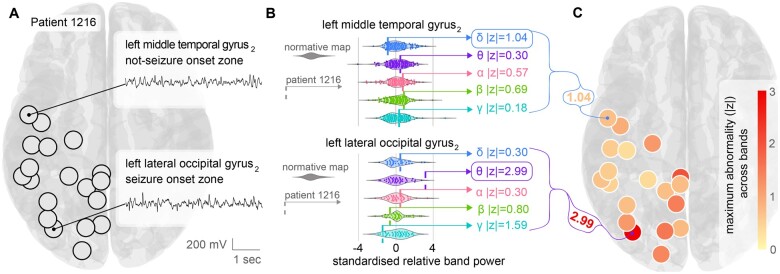
**Normative band power as a reference to detect abnormalities in individual patients.** (**A**) Visualization of the regions covered by the implanted electrodes in an example patient with epilepsy. 18 of the 128 regions were sampled by the electrode contacts in this patient (black circles). Time series from two example regions are shown that are without obvious epileptiform activity (inset). One example region (left lateral occipital gyrus 2) was the seizure onset zone in this patient. (**B**) Relative band power for each of the two regions, across each frequency band is plotted for the normative data (coloured violin plot; each point is a normative participant). Data are standardized (mean subtracted and divided by standard deviation). Relative band power *z*-score for Patient 1216 is plotted as a vertical dashed line on the same scale. The *z*-scores indicates that the left middle temporal gyrus is normal in all frequency bands (maximum absolute *z* = 1.04). The left lateral occipital gyrus is more abnormal in theta (maximum absolute *z* = 2.99) and gamma (absolute *z* = 1.59). (**C**) Maximum absolute *z*-score for each region plotted for the patient. Larger values indicate greater abnormality in any frequency band.

We use an example patient to illustrate the process. Patient ID 1216 had electrode contacts placed in the temporal, parietal and occipital lobes. Those electrodes were localized to corresponding regions of interest (black circles in Fig. 2A). We also show the interictal EEG time series of two example contacts in two different regions in Fig. 2A. The first region is the left middle temporal gyrus 2 (LMTG2), which is far away from the seizure onset zone in this patient. The second region is the left lateral occipital gyrus 2 (LLOG2), which is the seizure onset zone as determined by the presurgical evaluation.

On visual inspection, the two time series are not qualitatively different. However, following extraction of relative band power from the 70-s interictal recording for each of the two regions, and subsequent standardization to the normative distributions in each frequency band (violin plots in Fig. 2B), the LLOG2 region showed substantial deviations, particularly in the theta band (absolute *z*-score of 2.99). In contrast, the LMTG2 region did not display any strong deviations in any frequency band (≲1 SD away from the normative distributions).

We repeated the procedure of *z*-scoring all frequency bands in all regions of interest relative to the corresponding normative distributions, for example Patient 1216. It is conceivable that different frequency bands are abnormal in different regions and participants (Supplementary material). Therefore, to summarize these *z*-scores across frequency bands, we used the maximum absolute *z*-score as a measure of the regional level of aberration (Fig. 2C). Taking the maximum essentially summarized the level of interictal band power abnormality while allowing for region and participant-specific differences in terms of the frequency band. In this example patient, it is visually clear from Fig. 2C that the level of abnormality is highest in the LLOG2 region, but other occipital regions also presented with a high level of abnormality.

### Interictal band power abnormality distinguishes epileptogenic tissue

We next postulated that our measure of interictal band power abnormality of a region may serve as a marker of the region’s epileptogenicity. We thus hypothesized that the surgical removal of regions with the greatest abnormalities would be associated with postoperative seizure freedom. In contrast, if abnormal regions remain after surgery, we expect to see persistent seizures after surgery. To address this hypothesis, we retrospectively identified which regions were resected by surgery and compared the level of abnormality between surgically resected and spared regions.

**Figure 3:**
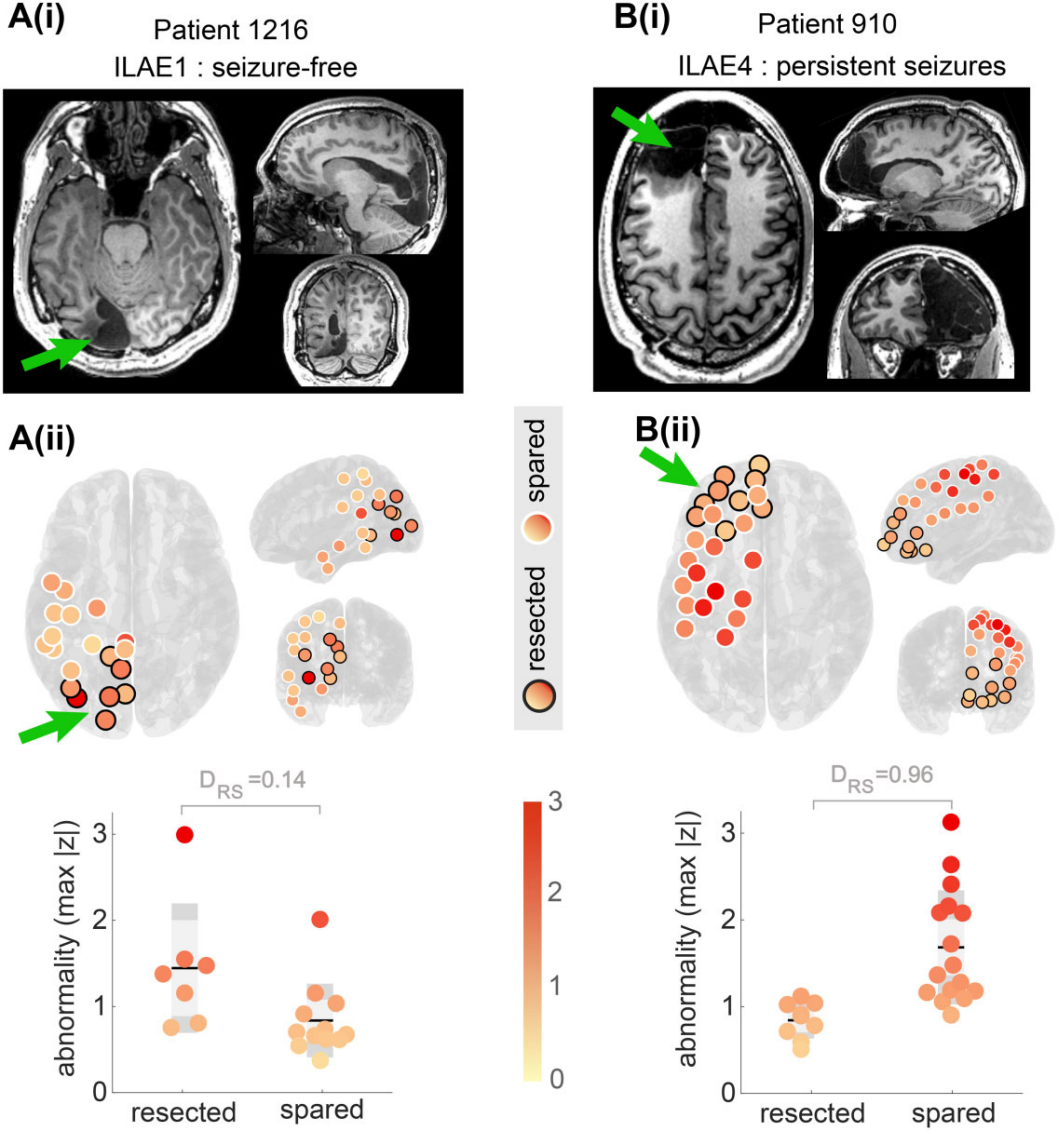
Interictal band power abnormality as a marker of epileptogenic tissue in two example individual patients. [**A**(i) and **B**(i)] Postoperative T_1_-weighted MRI scans showing the location of the resection as indicated by the green arrow. [**A**(ii)] Replication of the patient in Fig. 2 with the regions that were later surgically resected circled in black. Non-resected regions are circled in white. A direct comparison and quantification in the lower panel shows resected regions to be more abnormal than spared. Each data-point is a separate region. This patient was seizure free after surgery (ILAE1). [**B**(ii)] Visualization of data from a second patient with a frontal lobe implantation. Multiple abnormal regions were present outside the resection and spared by surgery. This patient had had continued postoperative seizures (ILAE4). In both patients, the $D_{RS}$ metric quantified the difference between resected and spared regions in terms of their abnormality.

The example Patient 1216 in Fig. 3A(i and ii) is the same patient shown in Fig. 2. The LLOG2 region was resected, along with other occipital regions. It is visually apparent that the resected regions [circled in black in Fig. 3A(ii)] appear substantially more abnormal than regions that were spared by surgery in this first example patient. The lower panel of Fig. 3A(i) quantifies the difference between the resected and spared regions using the $D_{RS}$ metric that quantifies the Distinguishability of the Resected and Spared regions in an individual patient.^23,^^28,^^29^$D_{RS}$ values close to zero indicate that resected regions are more abnormal than spared regions in that individual patient. In contrast, if $D_{RS}$ is close to 1, then spared regions are more abnormal than resected regions. A $D_{RS}=0.5$ indicates that the resected and spared regions are indistinguishable in terms of the level of interictal band power abnormality. Example Patient 1216 has a $D_{RS}=0.14$ [Fig. 3A(i)], indicating that regions removed by surgery were typically more abnormal than regions spared by surgery. This patient was subsequently seizure free on follow-up.

Interictal band power abnormalities of a second example patient (ID: 910), derived using the same processing and normative analysis, are presented in Fig. 3B(i and ii). This patient had an anterior frontal lobe resection. Their resection involved the removal of areas with normal interictal band powers ($\left|z\right|≲1$), while highly abnormal regions remained in more posterior parts of the frontal lobe. Analysis using $D_{RS}$ confirms this finding with $D_{RS}=0.96$, indicating that almost all spared regions were more abnormal than those resected. This example patient continued to have persistent postoperative seizures.

**Figure 4:**
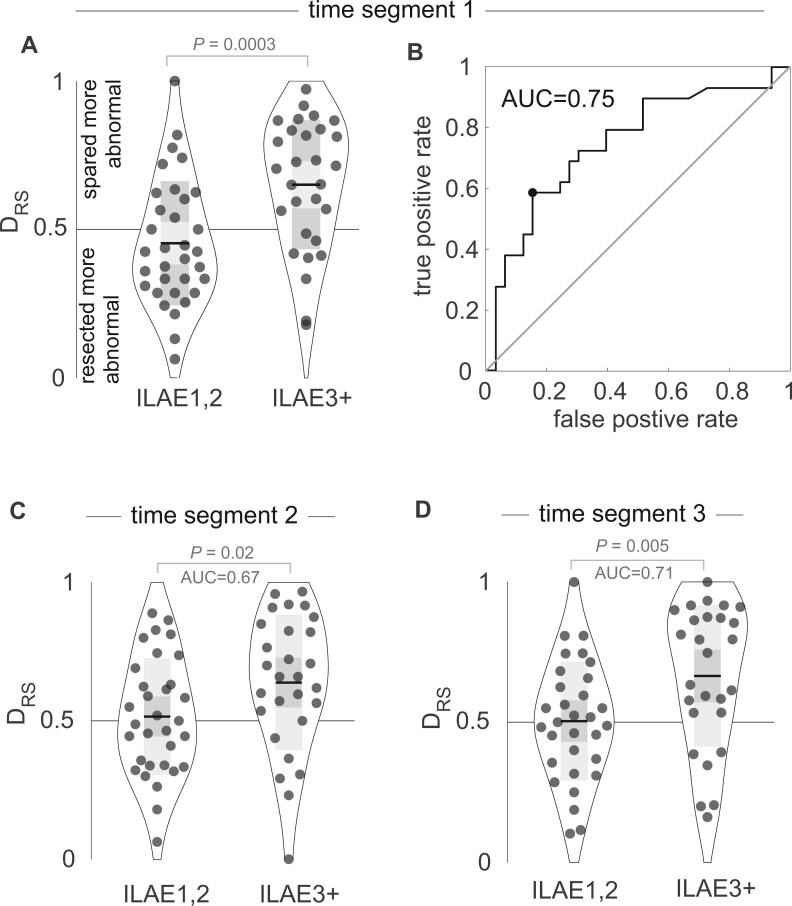
**Interictal band power abnormality distribution in resected versus spared tissue explains postsurgical seizure freedom.** (**A**) The $D_{RS}$ values, which indicate whether resected regions were more abnormal than spared regions, for each patient separated by outcome group. At a group level, the resected regions were more abnormal than spared regions in ILAE3+ patients, with substantially and significantly higher $D_{RS}$ values. Each point is an individual patient, black horizontal line indicates the mean, grey box indicates the standard deviation. (**B**) Using $D_{RS}$ as a binary classifier with a receiver operator characteristic curve (ROC) allows a calculation of the area under the curve (AUC) = 0.75 to predict ILAE outcome class. (**C** and **D**) Replication of the findings in (**A**) using other data segments at least 4 h away from the first data segment.

The two patients presented in Fig. 3 indicate that the interictal band power abnormality measure may serve as a marker of epileptogenicity, and its ability to distinguish resected from spared tissue ($D_{RS}$) may subsequently be used to predict seizure freedom after surgery. In Fig. 4A and B we generalize those findings across a cohort of 62 patients, with each datapoint representing an individual patient. At a group level, patients with persistent seizures (ILAE3+) had substantially and significantly greater $D_{RS}$ values than those who were free of disabling seizures (ILAE1,2) (right-tailed *t*-test *P* = 0.0003, *t* = −3.6, AUC = 0.75, see Fig. 4A and B). Furthermore, $D_{RS}$ values of patients with persistent seizures were substantially and significantly greater than 0.5, suggesting that abnormal regions were spared by surgery in ILAE3+ patients (*P* = 0.0003, *t* = 3.8, right tail *t*-test). Values of $D_{RS}$ for ILAE1,2 patients were not significantly less than 0.5 (*P* = 0.129, *t* = −1.15, left tail *t*-test). Taken together, these group-level findings suggest that regions with interictal abnormalities remain after surgery in patients with persistent postoperative seizures. Furthermore, the distinguishability between the resected and spared abnormality (i.e. $D_{RS}$) can discriminate between surgical outcome groups with AUC = 0.75.

In contrast, when only using the maximum relative band power in all 62 patients without scoring it against the normative map, patients with persistent seizures (ILAE3+) were not distinguishable from seizure-free patients in any individual frequency band (ILAE1,2) (δ AUC = 0.57 *P* = 0.28, θ AUC = 0.43 *P* = 0.25, α AUC = 0.39 *P* = 0.19, β AUC = 0.52 *P* = 0.78, γ AUC = 0.45 *P* = 0.70). This result highlights that it is indeed the abnormality relative to the normative map that contains information on epileptogenic tissue, rather than band power *per se*.

Finally, for clinical translation, it is also important to assess the robustness of our finding towards the exact interictal segment used. We chose two additional segments of interictal data in the 62 patients, where possible, separated by at least 4 h and at least 2 h away from seizures. Repeating the analysis on these two additional segments showed that $D_{RS}$ performed similarly well in discriminating between surgical outcome groups (AUC = 0.67, *P* = 0.02 and AUC = 0.71, *P* = 0.005; Fig. 4C and D).

### Robustness of findings

In this section, we assess the robustness of our results to various choices of parameters and factors that may influence our abnormality measure $\max\left(\left|z\right|\right)$. We first demonstrate the robustness of our results towards different parcellation schemes, frequency cut-offs, window sizes, segment length and normative outliers (Supplementary material). We further demonstrate adequate sampling of the resection by the parcellations (Supplementary material).

**Figure 5:**
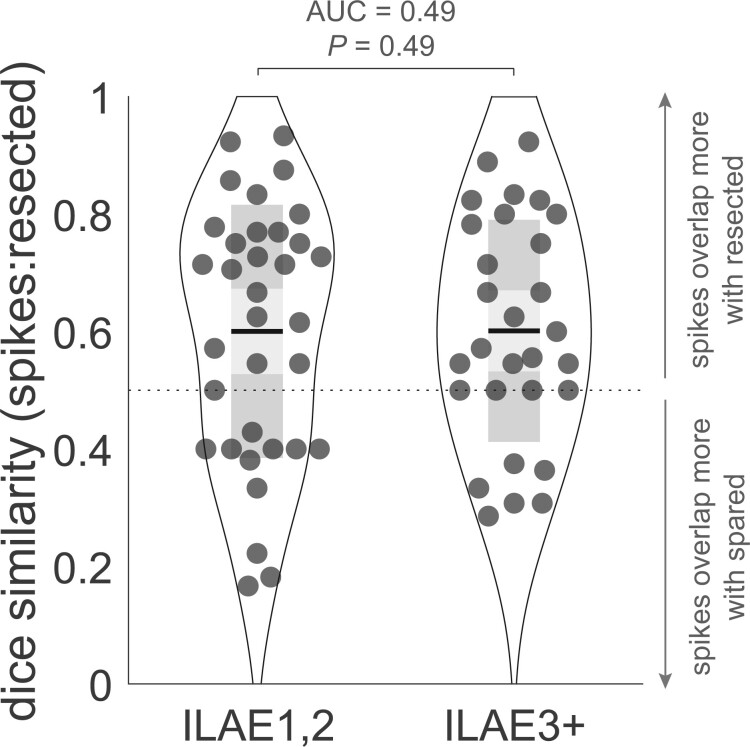
**Resection of interictal spikes does not explain outcome.** Data-points represent individual patients and indicate the dice overlap of regions containing contacts with interictal spikes and regions that were later resected. Although regions with spikes were more commonly resected (mean dice >0.5), the effect does not explain the outcome.

Finally, we compared the performance of our abnormality measure compared to interictal spikes as a marker of epileptogenic tissue. Interictal spikes are used as a clinical marker, and are often present for many patients. In Fig. 5, we investigate whether the resection of regions with spikes differentiates outcome groups. The dice similarity used in Fig. 5 captures the overlap between regions that were resected, and regions with spikes. Unsurprisingly, at a group level, patients had dice values >0.5, indicating that regions with spikes were more often resected. However, the dice similarity overlap does not differentiate outcome groups, unlike our abnormality based $D_{RS}$ measure (Fig. 4). Furthermore, if spikes were the main driver of our abnormality metric their presence in the time series would change $\max\left(\left|z\right|\right)$ substantially. However, in the Supplementary material, we demonstrate that this is not the case. Thus, we conclude that although interictal spikes may be present in patient EEGs, their resection does not distinguish outcome groups and they are not the main driver behind our results.

## Discussion

In this study we derived a normative map of relative band power across the brain using intracranial EEG. The use of normative baselines is commonplace in a wide range of neurology research; however, this approach is rare for invasive modalities such as intracranial EEG. By applying an intracranial EEG normative map in the context of epilepsy presurgical evaluation, we made several key contributions. First, we derived a normative map of interictal band power for different brain regions and frequency bands using the largest dataset so far. Second, we found that we can leverage this normative map to identify regional abnormalities within individual patients. Third, by overlaying abnormal regions with knowledge of resected tissue, we validated our identified abnormalities against surgical outcomes. Finally, we also demonstrated the robustness of our results to the choice of brain parcellation and iEEG segment.

Our normative map, inferred using intracranial EEG, has striking similarities to spectral profiles observed using other modalities such as MEG and scalp EEG.^9,^^10,^^12^ Some regional frequency-specific neocortical activity patterns are well known, including alpha in parietal regions and beta in motor areas.^30,^^31^ In complement to previous scalp EEG and MEG studies, our analysis also allows the investigation of deep brain subcortical structures with high spatial accuracy. Specifically, we report strong delta power in limbic structures including the hippocampus in agreement with one previous intracranial study.^13^ Interestingly, we also found strong delta power in anterior temporal and inferior frontal areas, as reported previously.^11^ Given the strong connectivity within limbic, anterior temporal and inferior frontal areas, including via the uncinate fasciculus, we suggest a potential structural underpinning for the spatial profiles observed in our normative maps. A future comparison of our normative map to a normative white-matter structural connectome could confirm this hypothesis for a given parcellation.

Few studies have used interictal intracranial recordings from multiple participants to infer a normative brain activity. An early study by Groppe *et al*.^15^ investigated data from 15 individuals and mapped spatial profiles of band power estimates. In agreement with our findings, they reported high beta power in motor areas and high theta in superior frontal areas (Fig. 1), amongst other spatial patterns. Perhaps most similar to our work is the study by Frauscher *et al*.,^13^ who created a normative map with intracranial data from 106 participants. The authors suggested that clinical EEGs could be compared to such a map to identify abnormal activity. Our study builds on this literature by creating an atlas from 234 participants and applying it to an independent sample of 62 patients with epilepsy.

After scoring the epilepsy cohort against the normative map, our goal was to detect abnormalities in interictal EEG activity that may help localize the epileptogenic tissue. To achieve this, we wanted to acknowledge the diversity of possible interictal abnormalities. Therefore, we extracted the maximum absolute abnormality in any frequency band. Our proposed $\max\left(\left|z\right|\right)$ measure is only one of several measures likely to be important for epileptogenic zone localization, and other dimensionality reduction techniques may be beneficial.^32^ Future studies should also investigate the band-specific abnormalities, and relate them to the participant-specific interictal activity patterns (e.g. spikes, slowing etc.) to aid interpretation. Here, we did not specifically investigate the relationship to particular interictal activity patterns, as we wanted to demonstrate a generalizable framework that can detect interictal abnormalities regardless of the specific nature, pattern, location or cause of the abnormality. However, it is conceivable that e.g. specific aetiologies are associated with specific patterns of interictal abnormality. We also did not control for other factors such as handedness, eyes open/closed or vigilance state etc. due to unavailability of this information in our retrospective study design. Future work should investigate the influence of epileptiform activity, as well as various other factors known to affect band power in EEG.

Our study further contributes to a growing literature searching for preoperative imaging markers of the epileptogenic zone that predict postsurgery patient outcomes.^33–40^ In general, two main approaches can be used to identify preoperative markers. The first is to use an entirely data-driven approach. Typically, this strategy involves high-dimensional data and feature-selection methods.^27,^^41^ However, interpreting the selected features may be challenging. In the present study, we instead used a hypothesis-driven approach to identify abnormal regions, which we hypothesized would remain after surgery in patients with persistent seizures. Other studies using hypothesis-driven approaches suggested that removing hub regions may explain outcomes,^23,^^28^ while using clinical demographics along with imaging has also been suggested.^42,^^43^ Note, however, that our proposed measure of band power abnormality may only be a sensitive, but not specific marker of the epileptogenic zone, as band power abnormalities outside of this region may also arise as a functional consequence of the seizures/epilepsy, e.g. through propagation of abnormal activity patterns or compensatory mechanisms. Because the exact boundaries of the epileptogenic zone are unknown, even after surgery, it is difficult to determine which abnormal regions definitively fall outside of this region. Notably, though, not all channels with a high abnormality need to be removed to achieve postoperative seizure freedom [Fig. 3A(i)]. In future work, we expect that combining different approaches and biomarkers that are differentially sensitive and specific will yield a translatable and interpretable biomarker of epileptogenic tissue and provide optimal predictions for postsurgical seizure freedom.^44^

Our normative map approach for localizing epileptogenic tissue could complement current clinical analysis of intracranial EEG. Currently, one of the key parts of presurgical evaluation is localizing seizure onset. However, resecting the seizure onset zone may not lead to seizure freedom in cases where the seizure onset zone and epileptogenic zone only partially overlap.^6^ Furthermore, seizure onset data may not be readily available, the onset location may not be consistent^45^ or the onset pattern may be diffuse for some patients, making it challenging or impossible to conclusively localize the epileptogenic zone using only their seizure data. Thus, to complement this approach, clinicians also evaluate interictal intracranial EEG for abnormalities such as spikes^6^ and high-frequency oscillations,^46^ which may be biomarkers of the epileptogenic zone. As discussed previously, visual inspection of intracranial EEG may miss more subtle frequency changes in neural activity, especially activity that is normal in one region may be abnormal if observed in another. By comparing interictal intranial EEG band power to a normative map, our approach highlights less salient, region-specific aberrations, providing a complementary tool to the traditional visual inspection of ictal and interictal EEG. In our validation, we therefore also opted to compare to surgically resected tissue and subsequent surgical outcome, rather than comparing with, for example, seizure onset zone or irritative zone.

To demonstrate the clinical usefulness of our approach, we showed that the discrimination of surgical outcome groups was robust to the choice of the interictal EEG segment. However, this finding should not be mistaken for evidence that interictal band power abnormality remains stable over time; rather, it simply demonstrates that the predictive power of this measure is not sensitive to abnormality fluctuations on the cohort level. Nevertheless, there is known variability in interictal dynamics in patients with focal epilepsy. For example, both the rate and spatial patterns of pathological interictal events such as spikes^7,^^47,^^48^ and high-frequency oscillations^3,^^49^ fluctuate during intracranial recordings. Further, interictal band power changes over a range of timescales (see Panagiotopoulou *et al*.^50^ and references therein) and, as a result, band power abnormality also fluctuates over time (Supplementary material). Future work will investigate the magnitude and timescales of these fluctuations and determine whether they hold additional information about epileptogenic tissue. In particular, abnormalities may be more salient following presurgical perturbations such as antiepileptic medication reduction or sleep deprivation,^51^ as well as during patient-specific phases of circadian or multiday cycles.^47,^^48^ Our observation that the group-level effect (in predicting surgical outcome) is largely unaltered across different time segment most probably reflects the fact that our sampling in time is random in each patient. In other words, if we can find an ‘optimal’ segment in each patient for detecting their band power abnormalities, we expect our group effect to be even higher. Additionally, like other interictal features,^3,^^52^ temporal changes of abnormalities could also be related to variable seizure features such as seizure onset^53^ or evolution^54^ within the same patient. Investigating such relationships could reveal additional applications for band power abnormalities, such as predicting seizure features.

Our study has several strengths and limitations. One strength is the sample sizes for both the normative map and epilepsy surgery datasets, which are some of the largest reported in the literature on intracranial EEG. Furthermore, the availability of patient data from other modalities including preoperative MRI, CT and postoperative MRI allowed for accurate electrode localization and delineation of resections. The reproducibility of the normative map across parcellations, and its agreement with existing literature, is also a major strength, providing confidence in our findings. The study’s limitations include the retrospective design of the study and the single-site origin of the patient data. Additionally, data regarding the brain state of the patients at the time of recording were not included in the analysis. Future studies could investigate whether normative maps and outcome predictions are affected by underlying state changes such as rest, tasks or sleep.

Patients undergoing invasive monitoring for surgical evaluation are typically those with the most uncertainty around where to operate, and they subsequently experience poorer outcomes as a difficult-to-treat cohort. Therefore, new ways to use invasive intracranial data are sought after to inform and improve clinical decision making. We envisage, in future, a software tool containing a normative map to which patient data and planned resections are compared.^27^ Such a tool would integrate other abnormality metrics from additional modalities including scalp EEG, MEG or MRI^18^ and make predictions of patient outcomes using advanced computational models of brain dynamics.^40,^^55^ Our findings pave the way to the use of normative intracranial baselines for clinical abnormality identification in epilepsy and beyond.

## Supplementary Material

awab380_Supplementary_Data
