# Supplementary material for: Normative brain mapping of interictal intracranial EEG to localize epileptogenic tissue
Source: Brain. 2022 Jan 24;145(3):939–49. doi: 10.1093/brain/awab380 (PMC9050535; doi:10.1093/brain/awab380)
Supplement: awab380_Supplementary_Data [file awab380_supplementary_data.zip › brain-2021-00988-File008.pdf]

Supplementary Material: Normative brain mapping of interictal intracranial EEG to localise epileptogenic tissue

Peter N Taylor[1,2,*,†], Christoforos A Papasavvas[1], Thomas W Owen[1], Gabrielle M Schroeder[1], Frances E Hutchings[1], Fahmida A Chowdhury[2], Beate Diehl[2], John S Duncan[2], Andrew W McEvoy[2], Anna Miserocchi[2], Jane de Tisi[2], Sjoerd B Vos[2], Matthew C Walker[2], Yujiang Wang[1,2,*,†]

[†]These authors contributed equally to this work.

Author affiliations:

1. CNNP Lab (www.cnnp-lab.com), Interdisciplinary Computing and Complex BioSystems Group, School of Computing, Newcastle Helix, Newcastle University, NE4 5TG, UK

2. UCL Queen Square Institute of Neurology & National Hospital for Neurology and Neurosurgery (NHNN), Queen Square, London WC1N 3BG, UK

*Correspondence to: Peter Taylor & Yujiang Wang

Full address: Urban Science Building, Newcastle Helix, Newcastle upon Tyne, UK

Email: peter.taylor@newcastle.ac.uk yujiang.wang@newcastle.ac.uk

Running title: Normative iEEG mapping and epilepsy

Keywords: cortical localization, EEG, epilepsy surgery, epileptogenic zone, intracranial electrodes

Supplementary analysis 1: the impact of interictal spikes

In the following analyses we investigate the impact of interictal spikes on our abnormality measure max |z|. We perform two separate analyses.

Our first spike analysis is as follows. Frequently spiking channels were marked by the clinical team at NHNN (Queen Square) for the UCLH dataset. We investigated (a) if our abnormality measure is different between those regions with regular spikes and those regions without (b), if the resection of regions with regular spikes differed between outcome groups, as our abnormality metric (D_{RS}) does, and (c) if the dice similarity (overlap between spikes and resection) is correlated with our D_{RS} abnormality metric (which captures the difference between resected/spared abnormality).

Analysis (a). We first investigated, for the two example patients presented in the main manuscript, if abnormality (max |z|) differs between regions with/without spikes (figure S1A). The rationale being that if our algorithm is driven by spikes, then there will be a clear increase in abnormality in spiking regions. We see in both example patients that such an increase is not present. Indeed, for patient 1216 many of the regions with inter-ictal spikes were actually less abnormal than their non-spiking counterparts. From this analysis we conclude that regions with marked spikes are not necessarily more abnormal than regions without marked spikes in these two patients.

To quantify the difference in abnormality between regions with regular spikes (S) and regions with no spikes (NS) we devised the $D_{S:NS}$ measure. The $D_{S:NS}$ measure is analogous to the D_{RS} metric. Instead of quantifying the difference in abnormality between resected and spared regions it quantifies the difference between the regions with spikes and with no spikes. Difference is calculated as an AUROC as described in methods. A $D_{S:NS}$ value of 1 indicates all regions with spikes are more abnormal than regions without spikes.

The $D_{S:NS}$ values for all patients are presented below (Figure S1B). The values below one for the majority of patients support the statement that interictal spikes are *not* the sole or main driver of our abnormality measure.

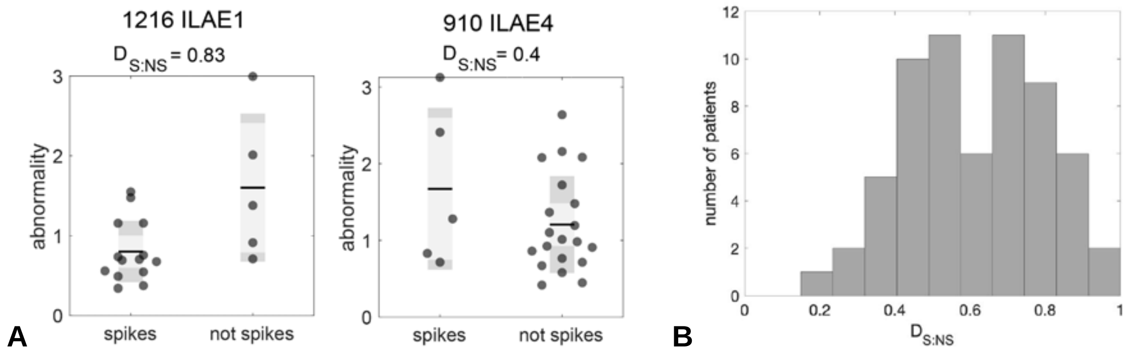

Figure S1: The relationship between regions with spikes and abnormality. **A** Data shown for the two example patients from figure 3 In the main text. Each data point represents an individual region. **B** Histogram of $D_{S:NS}$ values across patients. A value of 1 can be interpreted as all regions with spikes are more abnormal (max |z|) than all regions with no spikes. Almost all patients have values less than 1, with many around 0.5, suggesting the presence of abnormalities (max |z|) present in both regions with spikes, and regions without.

Analysis (b). We used the dice similarity as a measure of the overlap between those regions with spikes, and those regions which were later resected. Where the dice similarity equals one the resected and spiking regions match exactly, where the dice similarity equals zero the spikes overlap exactly with the spared regions. Dice equal to 0.5 represents chance-level overlap. If the removal (sparing) of interictal spikes was sufficient to explain the difference in patient outcomes, then the dice similarity would differ between outcome groups. We show in figure S2 below that dice similarity is not significantly different between outcome groups (p=0.49, auc=0.49). From this analysis we conclude that the resection of spike regions does not discriminate outcomes, unlike D_{RS} based on abnormality (Figure 4, main text). Unsurprisingly, dice similarity values are greater than 0.5 in most patients suggesting that regions with spikes were more commonly removed.

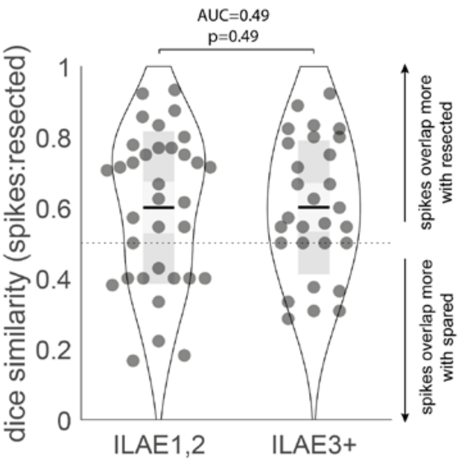

Figure S2: Overlap of regions with spikes and regions which were later resected, quantified using the dice similarity. Each datapoint is an individual patient. High values greater than 0.5 represent patients in which regions with spikes were resected, whereas low values less than 0.5 represent patients in which regions with spikes were spared.

Analysis (c). We investigated if there is a relationship between abnormality-based D_{RS} and spike-based dice similarity. We find no significant association between these two metrics in either outcome group (red and black data points represent individual subjects), or across our cohort as a whole (Figure S3). Thus we conclude that D_{RS}, based on abnormality, is not merely reflecting spike overlap with resection.

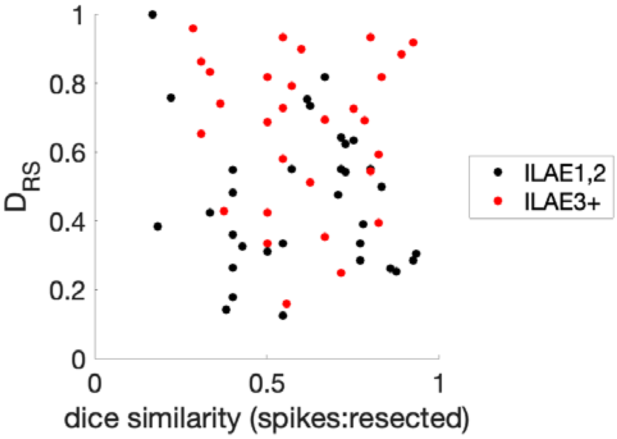

Figure S3: no relationship between D_{RS} (y axis) and dice similarity measure of overlap between regions with interictal spikes and resection (x axis). Each datapoint is an individual patient.

Our second analysis investigates the impact of additional spikes on our abnormality measure (max |z|).

We took the 70 second example time series from an electrode contact placed in the left middle temporal gyrus (part of which is shown in figure 2a in the main text) and artificially added simulated spikes using the approach proposed by Hu *et al*. 2020 (with the spike template derived from Kural *et al*. 2020). After adding simulated spikes we recalculated the relative band power of the modified time series, then computed the abnormality (max |z|). In the process of adding spikes two parameters must be chosen: (i) the number of spikes added (ii) the amplitude of the spike. These parameters are scanned in the plot below. We find that for frequent spikes (9 per minute, 15% of seconds) of very high amplitude (200 mV, equating to 8x standard deviation of the mean of the time series) there is a small change in our abnormality measure (max |z|), an increase of 0.2. This increase is encouraging as we would indeed consider the time-series to be more abnormal, since we have added spikes. Also encouraging however is that the increase is by only 0.2, which does not explain some of the very large abnormalities present in our cohort (see example patients in figure 3 of manuscript with abnormalities >2). We therefore conclude that although the addition of interictal spikes, just like any modification of the time series, will alter the PSD and subsequent max(|z|) the effect is relatively small.

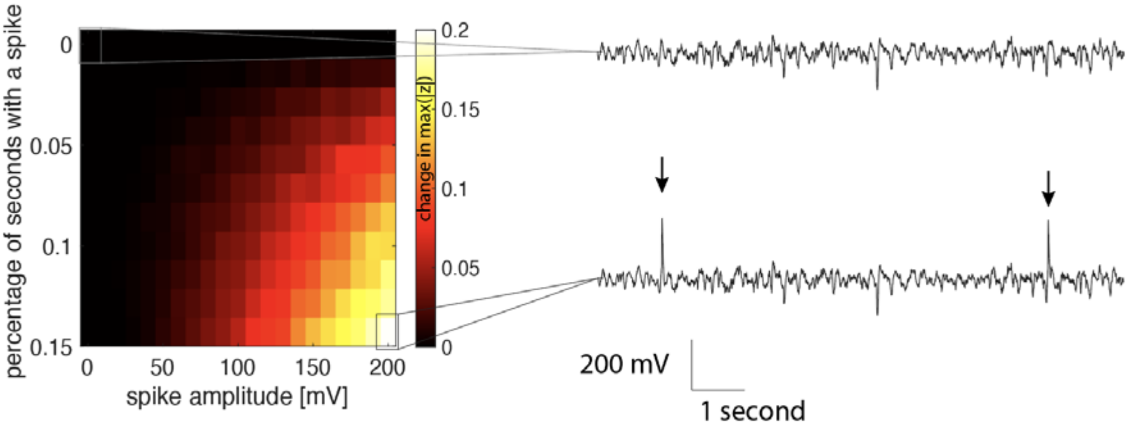

Figure S4: The change in max |z| is plotted for the addition of simulated spikes added to the time series of a region for different amplitudes and frequency (spike rate per minute). For spikes of zero amplitude, or zero frequency, the max |z| is unchanged (black colours in the left plot and top time

series on the right plot). For higher amplitude spikes (200mV) which occur frequently (9 per min, 15%) - lower time series - the max |z| changes by 0.2.

Our findings with the addition of simulated spikes further align with those from the study by Hu et al (2020) who also added simulated spikes to data to investigate their impact. The authors of that study concluded “Simulated spikes in healthy control EEG did not alter network strength or structure.”

Summarising these two separate analyses we conclude that although interictal spikes can alter a PSD and subsequent max(|z|) value, such spikes are not the only, or primary source driving our results.

Hu, D.K., Mower, A., Shrey, D.W. and Lopour, B.A., 2020. Effect of interictal epileptiform discharges on EEG-based functional connectivity networks. *Clinical Neurophysiology*, *131*(5), pp.1087-1098.

Kural, M. A., Duez, L., Hansen, V. S., Larsson, P. G., Rampp, S., Schulz, R., ... & Beniczky, S. (2020). Criteria for defining interictal epileptiform discharges in EEG: A clinical validation study. *Neurology*, *94*(20), e2139-e2147.

Supplementary analysis 2: the choice of frequency cut-off

Although many previous EEG studies use typical frequency bands (delta, theta, alpha, beta and gamma), their definition is not always consistent between studies. Here we investigate if our findings are sensitive to subtle changes in the boundary definitions for each band. Table SA1 below shows three examples using different frequency band cut-offs. Row one replicates the original results in the main manuscript. Rows two and three show the findings with slightly different cut-offs. We report that our findings are broadly similar and conclude to *not* be sensitive to subtle changes.

Table SA1: using slightly different cut-offs for each of the major frequency bands leads to similar results.

**Band1**	**Band2**	**Band3**	**Band4**	**Band5**	**AUC**	**P**
1-4	4-8	8-13	13-30	30-80	0.75	0.0003
1-3.5	3.5-7	7-12	12-28	28-70	0.77	0.00008
1-5	5-10	10-15	15-35	35-80	0.7137	0.0014

Supplementary analysis 3: Consistency using different window size & segment duration

In the main manuscript we used a window size of two seconds for Welch's method, over a segment of 70 seconds of data. Here, we investigated the consistency of our results when using different choices for these parameters. Figure S5 shows our findings to be broadly consistent and robust to the choice of window size and segment length. Note that the middle panels in figure S5 are identical and represent the parameter values used in the main manuscript.

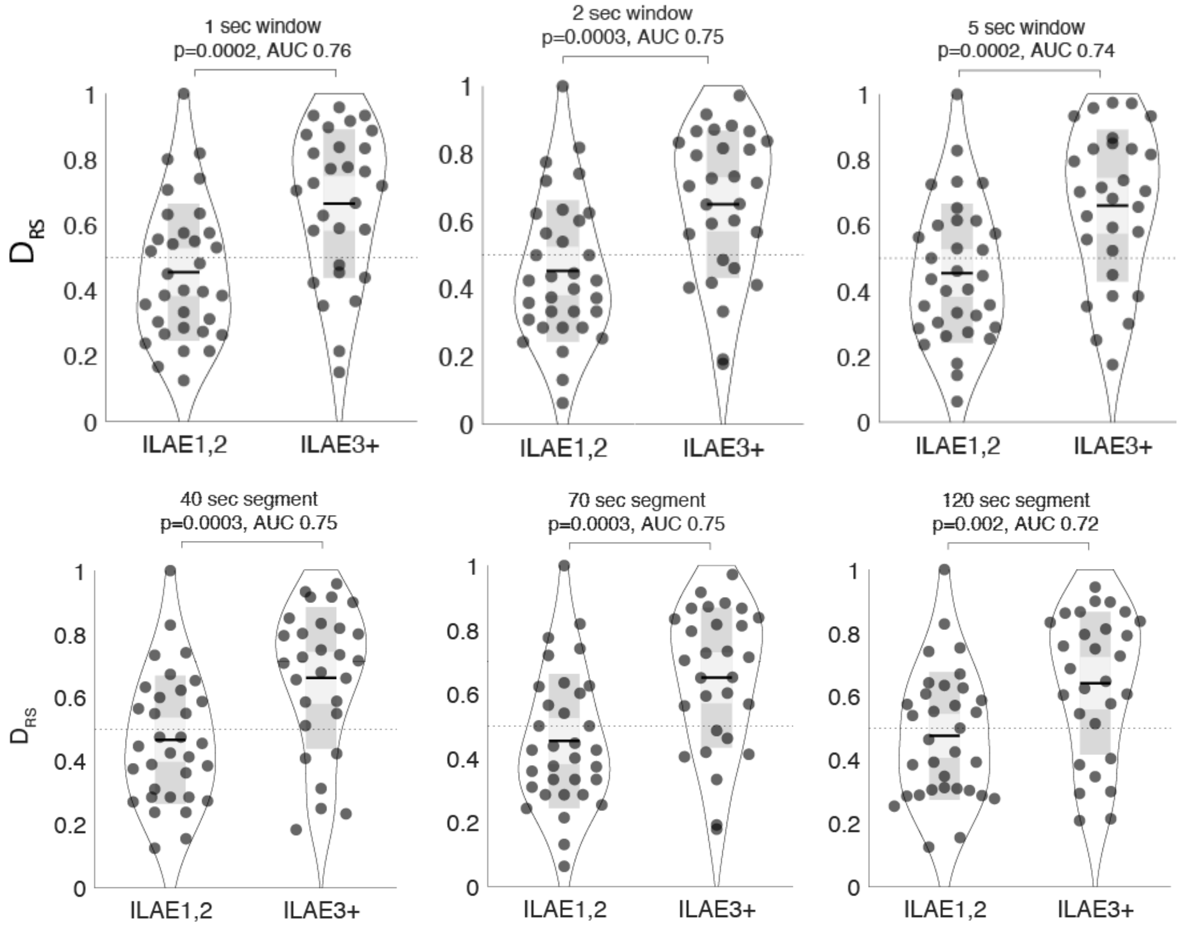

Figure S5: Robustness of results to choice of window or segment length

Supplementary analysis 4: Consistency across parcellations

In the main manuscript we used a parcellation with 128 regions of interest. Here we demonstrate the consistency of our result across alternative parcellations with different resolutions (figure S6). Both the normative map and the difference between ILAE1,2 and ILAE3+ groups are similar across all parcellations.

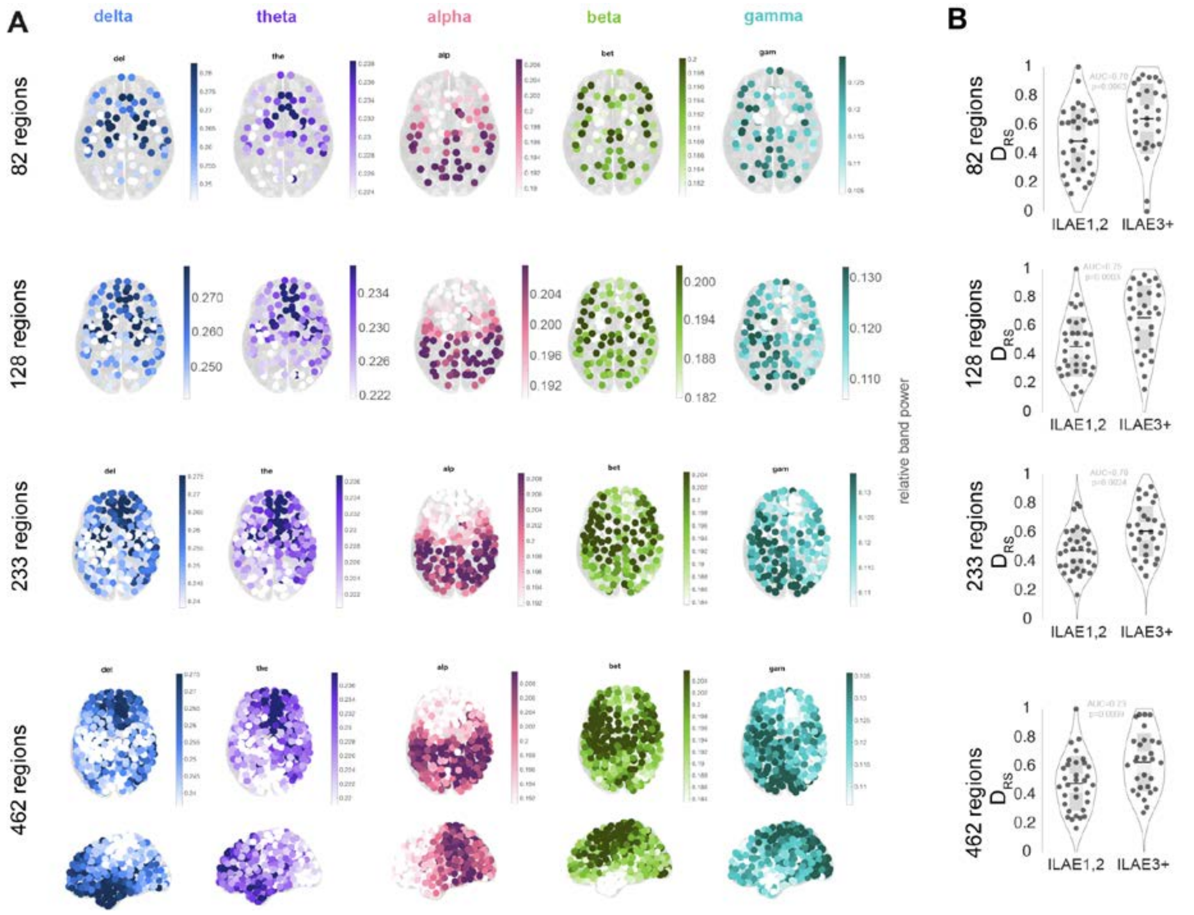

Figure S6: Replication of findings across parcellations **A.** Normative maps replicated for four different parcellations. The second row uses identical data to that shown in figure 1 in the main text. **B.** Replication of surgical outcome findings across parcellations.

Supplementary analysis 5: Lobe analysis of normative map

To facilitate quantification of the effect shown in figure 1 we present standardised data in figure S7. The standardisation procedure identifies, for each region, the amount by which that region is dominant in a given frequency band above and beyond other regions. Thus, by standardisation we identify which frequency is the strongest contributor to its activity, relative to other regions. Figure S7A shows the identified frequency for all regions, and figure S7B replicates this data for each lobe to assist visualisation. Clear spatial profiles can be observed with the strong parietal alpha activity shown in figure 1 brought through clearly in figure S7B. Through this visualisation other spatial variations in band power are distinctly visible, for example strong beta in frontal motor areas (green), theta in superior frontal areas (purple), and delta in temporal areas (blue) amongst others.

The regional differences in the normative map are visually apparent. To quantify and summarise these spatial variations we show in figure S7C the average relative band power within a lobe for each frequency band. Each lobe has distinctly different profiles and contributors, underscoring the substantial regional variations in band power.

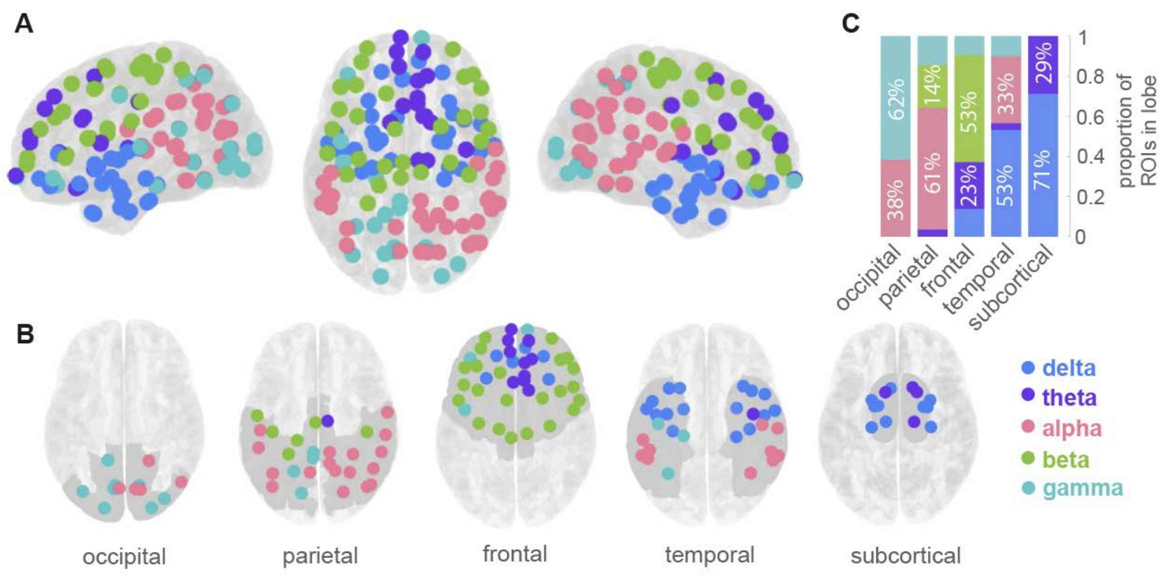

Figure S7: Standardised relative band power, with the maximum frequency band plotted. Standardisation (mean subtract, divide by standard deviation) across regions within each independent frequency band allows all panels in figure 1 of the main text to be placed on the same scale. Panel **A** shows the frequency band which is highest after the standardisation procedure. Note the pink regions in parietal areas which are due to the strong parietal alpha shown in figure 1 of the main text. **B** Replication of panel A showing lobes individually to facilitate visualisation. **C** Proportion of regions within a lobe which are dominant after standardisation for a given frequency. Note that 61% of parietal regions are dominant in alpha activity, thus quantifying the observations in panels A,B and figure 1 in the main text.

Supplementary analysis 6: spatial distribution of electrode coverage

Implantation of electrodes can differ between all subjects in terms of the location and number of regions covered. In this analysis we show that the coverage is broadly similar between datasets (Figure S8).

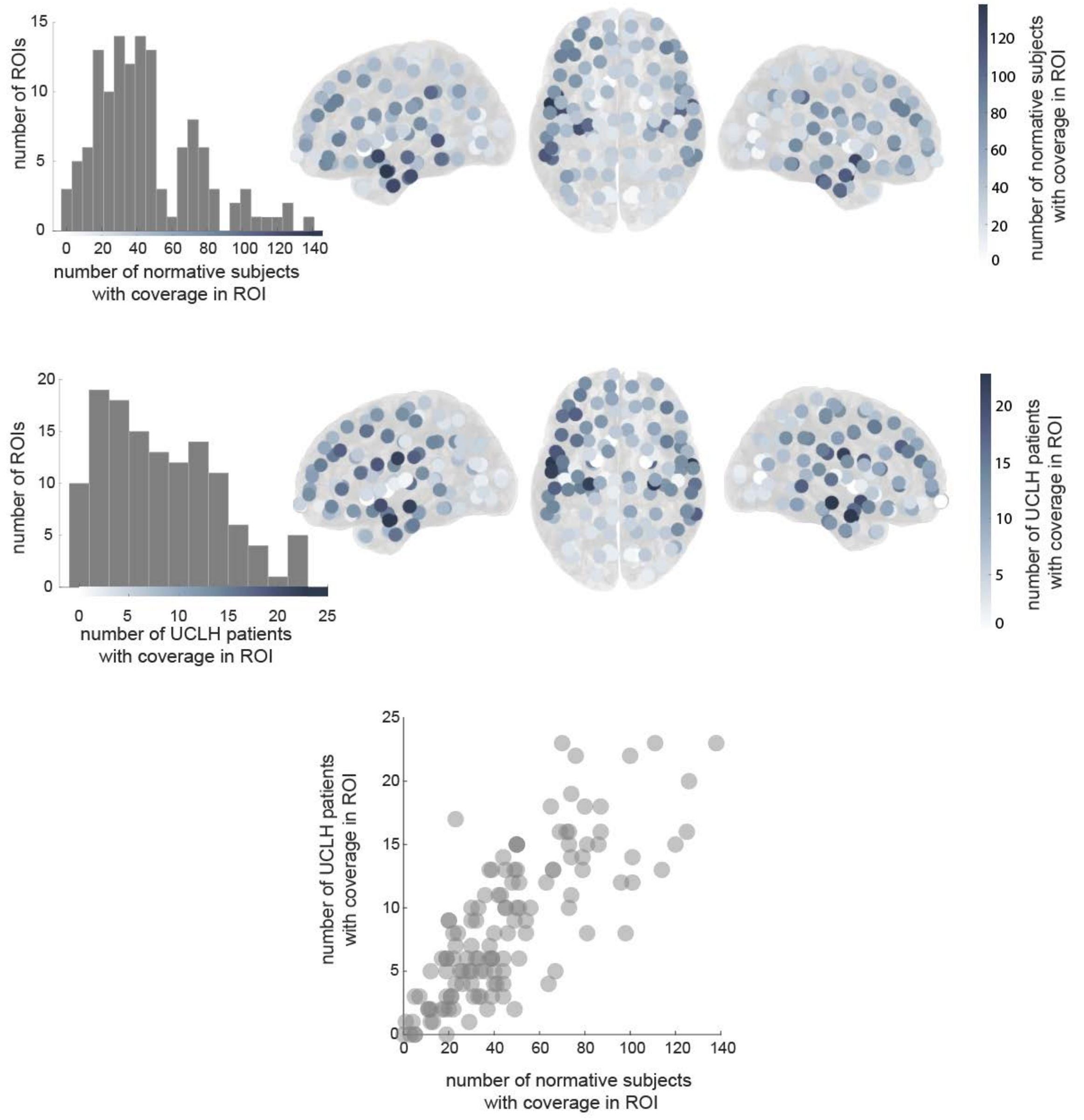

Figure S8: coverage of brain regions in the normative dataset (top panels) and the UCLH patient dataset (middle panels). Lower panel scatter plot shows high similarity in the spatial distribution of coverage between the datasets; each datapoint is a region.

Supplementary analysis 7: sensitivity to outliers in normative map

In the main manuscript we estimate abnormality in a patient's region as the absolute number of standard deviations (SD) from the mean of the same region in the normative data. However, if the normative data contains outliers they may bias the estimation of the mean in a disproportionate manner. As an alternative in figure S9, we replicate our analysis using the median and median absolute deviation (MAD). We find similar results in both techniques (Pearson rho = 0.92, figure S9).

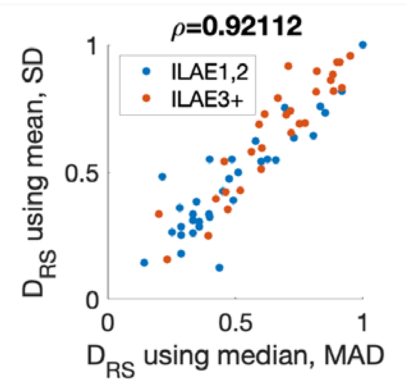

Figure S9: D_{RS} using median & median absolute deviation (MAD) is very similar to D_{RS} using mean & standard deviation (SD).

Supplementary analysis 8: Individual patient's max |z| abnormality

Here we investigated if particular frequency bands selected as most abnormal (highest max |z|) were more commonly resected regions in patients. In figure S10 we show the frequency bands selected as maximally abnormal for each region as a proportion of all resected regions. As an example, patient 1038 had 7 regions resected. Six of the 7 were most abnormal (had max |z|) in beta (shown as 86% green), whilst one of 7 regions was most abnormal in delta (shown as 14% blue). There is no clear consistency across subjects regardless of outcome group in this individual patient analysis.

Following the previous individual patient analysis in figure S10, figure S11 shows a group analysis which combines all regions for all patients dichotomised by outcome and resected/spared. We observe no substantial difference between any of the four groups represented in figure S11. We thus conclude our results are not driven by one specific frequency band.

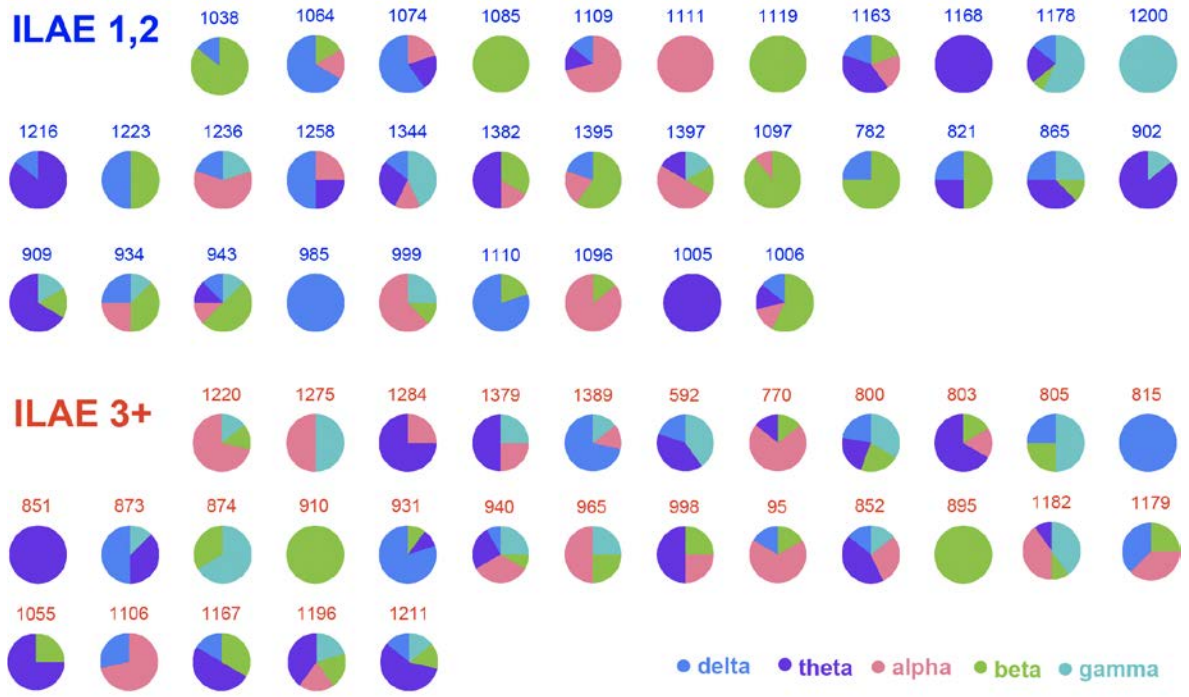

Figure S10: The frequency band found to be most abnormal (have the max |z|) in the resected regions plotted as a pie chart per subject. As an example, patient 1038 had 7 regions resected. Six of the 7 were most abnormal in beta (shown as 86% green), whilst one of 7 was most abnormal in delta (shown as 14% blue). Each chart is an individual patient, the number above the chart is the patient identifier referred to in table S1.

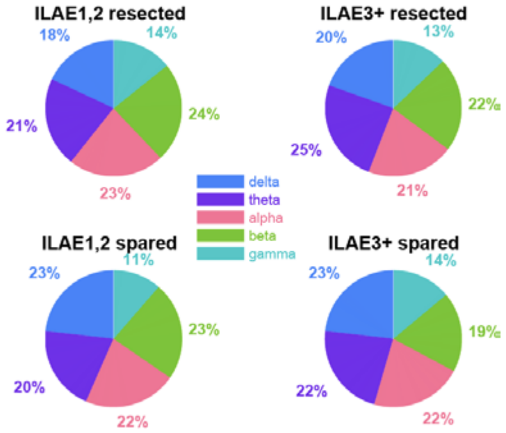

Figure S11: across all subjects the regions' band selected as max |z| is approximately evenly distributed across delta, theta, alpha, beta, and gamma.

Supplementary analysis 9: Volumetric size of the resection

In this analysis we investigate the size of the resection and the size of the regions of interest for a given parcellation (figure S12). Region volumes (top row) reported below are from the MNI space atlas, and not specific to any individual patient. We find that most regions in all parcellations have smaller volumes than the average resection volume, and hence should produce adequate sampling. However, this will be subject and location-dependent, and in theory parcellation 4 should provide the best sampling of the resected tissue, but will also provide the least confidence in each ROI normative distribution.

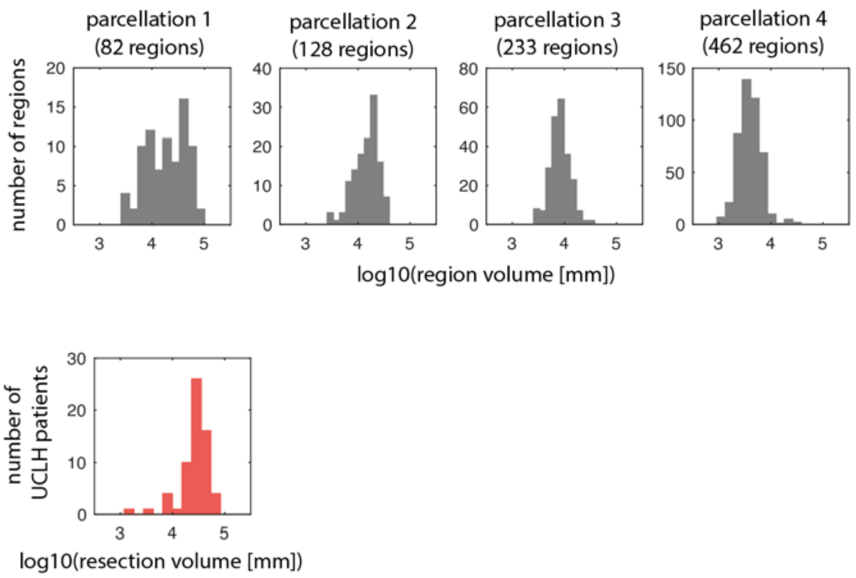

Figure S12: Upper panels, volumetric size of each region in each parcellation. Lower panel, volumetric size of the resection in each patient.

Supplementary analysis 10: Consistency of D_{RS}

In the main text, we presented a consistent AUC regardless of the different time segments we used for each patient. Here we present additional results that clarify the interpretation of the main results. The AUCs presented in the main text measure a group-level effect of D_{RS} being different between surgical outcome groups. However, the consistency of the AUCs across time segments should not to be interpreted as the D_{RS} or the abnormality remaining static over time. Rather, it only indicates that the group-level effect remains regardless of time segment. Below (Fig. S13), we show the actual D_{RS} values for each segment scattered against that of another segment for comparison (each datapoint is a patient). While we can see an overall correlation between the time segments (indicating that the D_{RS} values do not change dramatically from one segment to the next), there is also an amount of temporal variability (as expected - see Discussion).

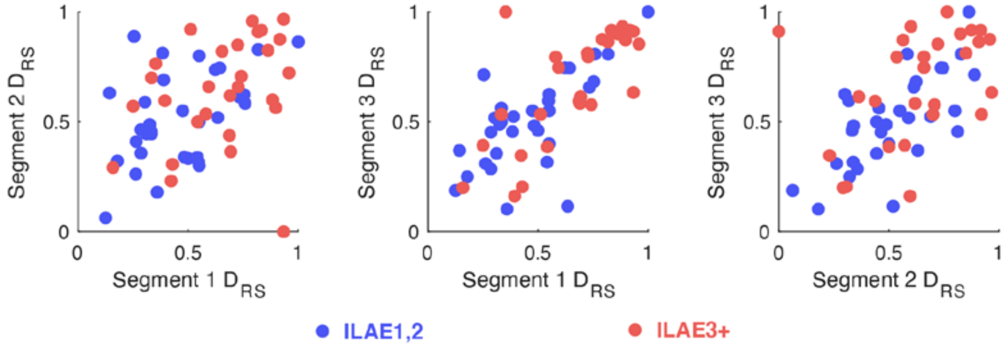

Figure S13: relating D_{RS} values across segments shows some variations, but with a sufficiently large effect size to discriminate outcomes. Each data point is an individual patient.

This result indicates that abnormality levels can fluctuate over time in each patient, and the fact that the AUCs remain similar across time segments reflects the random sampling of different time segments in each patient. In other words, there may be time periods where abnormalities become more salient in each patient, and if we could find and use such time periods *a priori*, then our group-level effect is also expected to increase.
