# Supplementary material for: Normative brain mapping of interictal intracranial EEG to localize epileptogenic tissue
Source: Brain. 2022 Jan 24;145(3):939–49. doi: 10.1093/brain/awab380 (PMC9050535; doi:10.1093/brain/awab380)
Supplement: awab380_Supplementary_Data [file awab380_supplementary_data.zip › brain-2021-00988-File009.pdf]

Table S1: patient data

ID	ILAE Outcome	DRS	Age	Sex	OP Type	Op Side	Op Pathology	Number Electrode contacts
782	1	0.33	28	M	T Lx	L	OTHER	54
821	1	0.63	26	F	T Lx	L	HS	46
865	1	0.13	28	F	T Lx	L	OTHER	52
902	1	0.25	30	F	T Lx	L	DUAL	63
909	1	0.33	26	M	T Lx	L	HS	26
934	1	0.33	29	F	O P Lx	R	OTHER	75
943	1	0.55	30	F	T Lx	R	FCD	32
985	1	0.30	35	M	T Lx	L	OTHER	58
999	1	0.55	29	M	F Lx	L	FCD	77
1006	1	0.55	28	F	F Lx	R	FCD	104
1064	1	0.26	31	M	T Lx	L	DNT	97
1074	1	0.31	52	M	T Lx	L	HS	32
1085	1	0.48	27	M	F Lx	L	FCD	84
1097	1	0.82	29	M	F Lx	L	GL	84
1109	1	0.18	32	F	T Lx	R	CAV	54
1111	1	0.39	25	F	T Lx	R	FCD	102
1119	1	0.62	32	M	F Lx	L	FCD	121
1163	1	0.75	27	F	F Lx	L	FCD	109
1200	1	0.73	24	F	T Lx	R	HS	68
1216	1	0.14	22	M	O P Lx	L	FCD	71
1223	1	0.43	56	M	F Lx	L	FCD	82
1236	1	0.76	21	M	T Lx	R	HS	79
1258	1	0.48	45	M	T Lx	R	DNT	71
1344	1	0.64	60	F	T Lx	R	OTHER	58
1382	1	0.26	29	F	T Lx	R		80
1395	1	0.54	32	M	T Lx	L		89
1397	1	0.50	31	F	T Lx	R	OTHER	73
1005	2	1.00	22	F	T Lx	R	HS	88
1038	2	0.29	44	F	T Lx	R	HS (EFS)	28
1096	2	0.38	30	M	F Lx	R		78
1110	2	0.55	24	F	T Lx	R	OTHER	34
1168	2	0.36	60	F	F Lx	L	FCD	94
1178	2	0.29	23	F	F Lx	L	OTHER	82
592	3	0.93	50	F	T Lx	R		53
770	3	0.58	25	F	P Lesx	L	FCD	73
965	3	0.79	32	M	P Lesx	R	FCD	89
1182	3	0.86	28	M	P Lesx	R	FCD	72
1196	3	0.33	42	M	T Lx	R	HS	24
1211	3	0.25	26	M	T Lx	R	OTHER	74
1284	3	0.82	21	M	F Lx	R	FCD	62
1379	3	0.74	30	M	T Lx	R		71
1389	3	0.59	46	F	F Lx	L		77
95	4	0.93	36	M	O P Lx	L	CAV	55
800	4	0.39	30	F	T Lx	L	HS	61
803	4	0.65	33	M	T Lx	R	NAD	52
805	4	0.73	32	F	T Lx	L	OTHER	62
852	4	0.54	45	M	F Lx	R	OTHER	63
873	4	0.69	31	F	T Lx	L	HS (EFS)	72
895	4	0.16	17	F	F Lx	L	DNT	96
910	4	0.96	23	M	F Lx	L	FCD	120
931	4	0.88	29	M	T Lx	L	HS (EFS)	54
998	4	0.69	31	M	T Lx	L	OTHER	22
1055	4	0.51	47	M	T Lx	R	OTHER	38
1106	4	0.43	27	M	F Lx	R	FCD	122
1167	4	0.92	39	M	P Lx	L	CAV	51
1179	4	0.73	45	F	T Lx	L	HS	57
1220	4	0.82	26	M	T Lx	L	OTHER	96
1275	4	0.42	38	F	T O Lesx	R	DNT	48
815	5	0.83	23	F	T Lx	L	OTHER	40
851	5	0.35	43	F	F Lx	L	OTHER	68
874	5	0.90	37	F	F Lx	L	FCD	67
940	5	0.69	24	M	T Lx	R	OTHER	72
